# A simple and fast ultrasonographic method of detecting enteral feeding tube placement in mechanically ventilated, critically ill patients

**DOI:** 10.1186/s40560-017-0249-5

**Published:** 2017-08-18

**Authors:** Wagner Luis Nedel, Mariana Nunes Ferreira Jost, João Wilney Franco Filho

**Affiliations:** Intensive Care Unit, Hospital Nossa Senhora da Conceição, Av. João XXIII, 525, 801E, São Sebastião, Porto Alegre, RS 91060-100 Brazil

**Keywords:** Enteral feeding, Mechanical ventilation, Ultrasound, Nasogastric feeding tube, Enteral nutrition

## Abstract

Abdominal X-rays, the diagnostic method for enteral feeding tube (EFT) positioning, are a source of irradiation for the patients and carry a potential risk of adverse effects. Data related to ultrasound (US)-guided EFT placement are scarce. We evaluated 41 patients with 41 EFT insertions with guidewire in place that was maintained until US examination. US detected 38 patients with proper positioning and 3 with inadequate positioning, with a sensitivity of 97% (95% CI 84.9–99.8%) and specificity of 100% (95% CI 19.7–100%). The assessment of EFT position through abdominal US is practical and safe, associated with satisfactory diagnostic accuracy.

## Background

Enteral nutrition (EN) is the feeding route of choice for critically ill patients with a functional gastrointestinal tract [1]. An adequate EN should deserve an enteral feeding tube (EFT) placement, frequently requiring an image confirming the correct position. Abdominal X-rays (AXRs), the gold standard diagnostic method for EFT placement, are a source of irradiation for the patients and carry a potential risk of accidental removal of devices, microbial dissemination, additional costs, and a delay in prompt image [2, 3]. Many procedures in intensive care units (ICUs) are performed under ultrasound (US) guidance [4], but data related to US-guided EFT placement in ICU patients are scarce [2, 3]. Therefore, we aimed to prospectively evaluate the effectiveness of US-guided correct EFT placement in mechanically ventilated (MV) ICU patients with a rapid, simple, and fast technique based on visualization of guidewire inside EFT. The secondary objective is to compare time to perform AXR and US to the correct diagnosis.

## Methods

We evaluated prospectively patients in invasive MV with EFT insertion on ICU. All EFTs were progressively inserted to the distance from the xiphisternum to the nose via the earlobe, with a guidewire facilitating tube insertion, maintained until US examination, as EFTs have radiopaque marks. Since the EFT is thin and soft, a guidewire was maintained post insertion to a better US visualization, seen as a hyperechoic image.

**Fig. 1:**
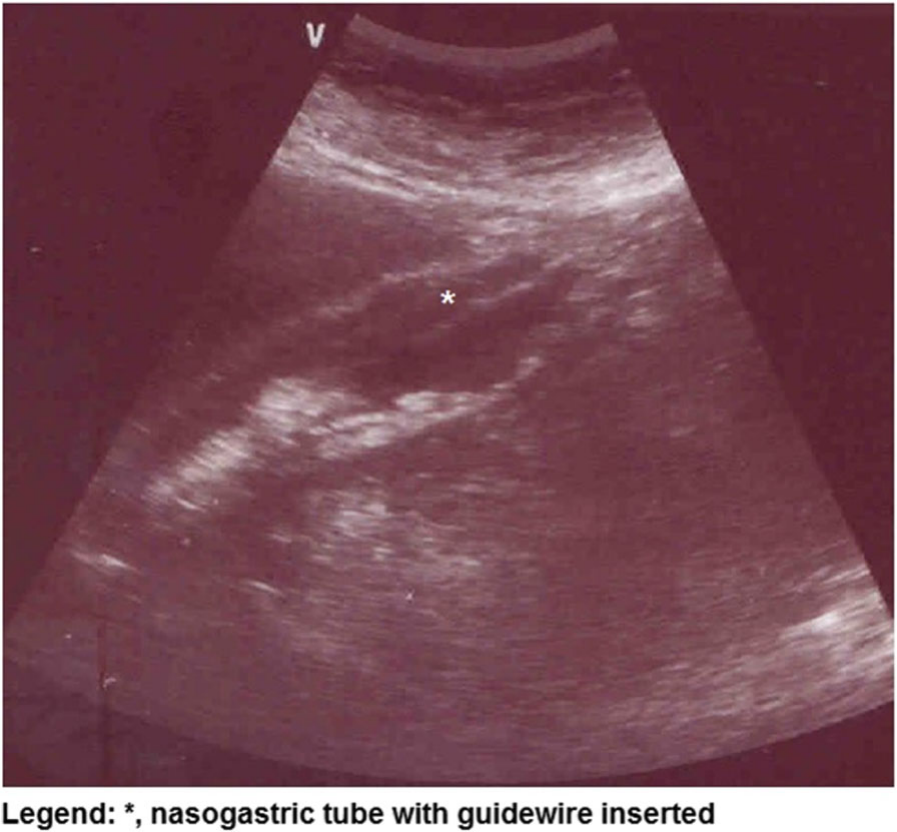
Gastric area and guidewire positioning

US was performed by non-assistant physician (M Turbo™ by Sonosite Fuji Film, Bothell, USA), and all AXRs were analyzed retrospectively in the end of study in a blinded fashion and prospectively by ICU team. The principal investigator (WLN) was an ICU physician with a minimum of 20 examinations experience in US EFT position technique. The patient is scanned with a 3-MHz curvilinear probe, performing abdominal examination with sagittal and transverse sweeps through the middle epigastric area, and if EFT was not visualized, the probe was oriented towards the left upper abdominal quadrant to visualize the gastric area, identifying both the digestive tract and the tube with the guidewire inside the digestive tract (Fig. 1). We did not record the precise gastric or duodenal location of EFT tip in this study, only if there was a proper positioning in the digestive system, instead of an inadequate esophageal, oropharyngeal or tracheobronchial tree positioning. Time between EFT placement and US diagnosis was compared with time between procedure and AXR diagnosis, defined as the time between implantation of the EFT and the release of the image to evaluate its positioning. Continuous variables are expressed as median and interquartile (IQR) range. The time taken by each method were compared by using Wilcoxon signed-rank test. Sensitivity, specificity, positive, and negative predictive values were calculated with 95% confidence intervals (CI). Statistical analysis was performed using SPSS version 20.0 (SPSS Inc., Chicago, USA). A value of *p* < 0.05 was considered to be statistically significant.

## Results

Forty-one patients (53% male, 14% surgical) were analyzed with 41 EFT insertions, in which the duration of the US presented a time of accomplishment of 90 s (45–167 s). The main clinical charts are expressed in Table 1. Thirty-nine patients presented a proper EFT positioning (95%), and in 2 patients, this was inadequate through the AXR. US evaluation detected 38 patients with proper and 3 with inadequate positioning (Table 2), with a sensitivity of 97% (95% CI 84.9–99.8%), specificity of 100% (95% CI 19.7–100%), positive predictive value of 100% (95% CI 88.5–100%), and negative predictive value of 66% (95% CI 12.5–98%). Median time between the installation of EFT and the diagnosis of EFT position by US was 46 min (20–163 min), while median time for AXR was 162 min (98–247 min), *p* < 0.0001.

**Table 1:** Main clinical charts

Variable	Median (IQR) or *n* (%)
Age	62 (48.5–68)
BMI	22 (19.2–28)
Charlson comorbidity index	3 (1–7)
Tracheostomy	5 (12)

*BMI* body mass index, *IQR* interquartile range, *n* number of patients

**Table 2:** Accuracy of diagnostic test

	AXR positive	AXR negative	Total
US positive	38	0	38
US negative	1	2	3
Total	39	2	41

*AXR* abdominal X-ray, *US* ultrasound

## Discussion

We were able to obtain a quick and fast imaging of the EFT placement in a great majority of MV critically ill patients. We confirmed the high sensitivity of this method in ICU patients, even when performed by a physician that do not have a formal graduation in US. This study adds new data about this topic, previously described in pediatric population [5] with posterior extension to adults, with similar results in few previous studies [2, 3], despite the technique used. Enteral feeding tube or nasogastric tube is generally detected visualizing through its acoustic shadow more than the tube itself, and our technique possibly are easier to perform. Our main objective in this study was to determine the proper position of the EFT in digestive tract, not analyzing the pre- or post-pyloric positioning, which, in the vast majority of patients, will not lead to better feeding acceptance or greater gastrointestinal tolerance [6].

This study has some limitations. Despite the shorter time for an accurate diagnosis of the probe positioning through US, this data should be interpreted with caution. Each ICU has distinct logistic capabilities, both for the diagnosis and for a faster onset of feeding, and therefore, the time to perform both exams may be quite different from that found in our study. These data, therefore, do not have external validity. Possibly in other scenarios, the time of diagnosis by the US is shorter than the one of the AXR, however quite different from the time differences found in this study. Radiography is still the standard method for confirmation of EFT placement, and the role of US as an alternative should be tested in prospective randomized studies, demonstrating how much sonographic applications decrease the necessity for radiographs and the rate of complications of EFT placement.

## Conclusion

The assessment of the proper EFT through abdominal US is practical and safe, with fast execution and possibly associated with satisfactory diagnostic accuracy.

## Abbreviations

AXRs: Abdominal X-rays
EFT: Enteral feeding tube
ICU: Intensive care unit
MV: Mechanical ventilation
US: Ultrasound
